# Beta‐amyloid 1‐42 monomers, but not oligomers, produce PHF‐like conformation of Tau protein

**DOI:** 10.1111/acel.12500

**Published:** 2016-07-12

**Authors:** Giusi Manassero, Michela Guglielmotto, Raluca Zamfir, Roberta Borghi, Laura Colombo, Mario Salmona, George Perry, Patrizio Odetti, Ottavio Arancio, Elena Tamagno, Massimo Tabaton

**Affiliations:** ^1^ Department of NeuroscienceUniversity of Torinovia Cherasco 1510126TorinoItaly; ^2^ Neuroscience Institute of Cavalieri Ottolenghi Foundation (NICO)University of TorinoRegione Gonzole 1010043OrbassanoTorinoItaly; ^3^ Unit of Geriatric MedicineDepartment of Internal Medicine and Medical Specialties (DIMI)University of GenovaViale Benedetto XV, 616132GenovaItaly; ^4^ Department of Molecular Biochemistry and PharmacologyIRCCS‐Istituto di Ricerche Farmacologiche ‘Mario Negri’Via Giuseppe La Masa19, 20156MilanItaly; ^5^ College of SciencesThe University of Texas at San AntonioOne UTSA CircleSan AntonioTX78249USA; ^6^ IRCCS San Martino‐ISTUniversity of GenovaViale Benedetto XV, 616132GenovaItaly; ^7^ Department of Pathology and Cell BiologyTaub Institute for Research on Alzheimer's Disease and the Aging BrainColumbia University630 West 168th Street, P&S 12‐420DNew YorkNY10032USA

**Keywords:** Alzheimer's disease, beta‐amyloid, hTau mice, MAPK, PHF, tau protein

## Abstract

The mechanistic relationship between amyloid β1‐42 (Aβ1‐42) and the alteration of Tau protein are debated. We investigated the effect of Aβ1‐42 monomers and oligomers on Tau, using mice expressing wild‐type human Tau that do not spontaneously develop Tau pathology. After intraventricular injection of Aβ1‐42, mice were sacrificed after 3 h or 4 days. The short‐lasting treatment with Aβ monomers, but not oligomers, showed a conformational PHF‐like change of Tau, together with hyperphosphorylation. The same treatment induced increase in concentration of GSK3 and MAP kinases. The inhibition of the kinases rescued the Tau changes. Aβ monomers increased the levels of total Tau, through the inhibition of proteasomal degradation. Aβ oligomers reproduced all the aforementioned alterations only after 4 days of treatment. It is known that Aβ1‐42 monomers foster synaptic activity. Our results suggest that Aβ monomers physiologically favor Tau activity and dendritic sprouting, whereas their excess causes Tau pathology. Moreover, our study indicates that anti‐Aβ therapies should be targeted to Aβ1‐42 monomers too.

## Introduction

A series of clues indicate that in Alzheimer's disease (AD), the accumulation of amyloid β (Aβ) in the brain is the primary and early event that induces neuronal degeneration, characterized by accumulation of conformational altered and aggregated Tau protein.

Aβ derives from the amyloid precursor protein through β site APP cleaving enzyme 1 (BACE1) and γ‐secretase processing that generates multiple C‐termini, most ending at residue 40 and 42. Aβ1‐42 aggregates more quickly and stably than Aβ1‐40. Aβ1‐42 polymerization is believed to occur in sequential phases: First Aβ monomers aggregate into soluble oligomers that then form insoluble oligomers, generating protofibrils and fibrils (Hubin *et al*., 2014). Soluble Aβ1‐42 oligomers constitute the more toxic form of the peptide (Bolmont *et al*., 2007; Nimmrich & Ebert, 2009; Guglielmotto *et al*., 2014). Monomers have been proposed to be involved, preferentially, in physiologic processes (Puzzo *et al*., 2011; Piccini *et al*., 2012).

How Aβ mediates alteration and aggregation of Tau is uncertain, although three major mechanisms have been proposed: (i) Aβ activates kinases that phosphorylate Tau (Hernández & Avila, 2010; Llorens‐Martín *et al*., 2014) altering its capacity to bind tubulin; (ii) Aβ alters the proteasomal degradation of Tau, increasing its concentration in free state (Oddo *et al*., 2009); (iii) Aβ intracellular aggregates have a nucleation effect on Tau (Guo *et al*., 2006; Bolmont *et al*., 2007). The third hypothesis is supported by the occurrence of Tau pathology in different types of cerebral amyloidosis (Holton *et al*., 2001; Giaccone *et al*., 2008).

In this work, given the relevance of soluble monomeric as well as oligomeric state of Aβ1‐42 in AD pathogenesis, we studied their role in altering the aggregation and conformation of Tau protein when injected in mice with a pure human Tau (hTau) background (Andorfer *et al*., 2003).

## Results

**Figure 1:**
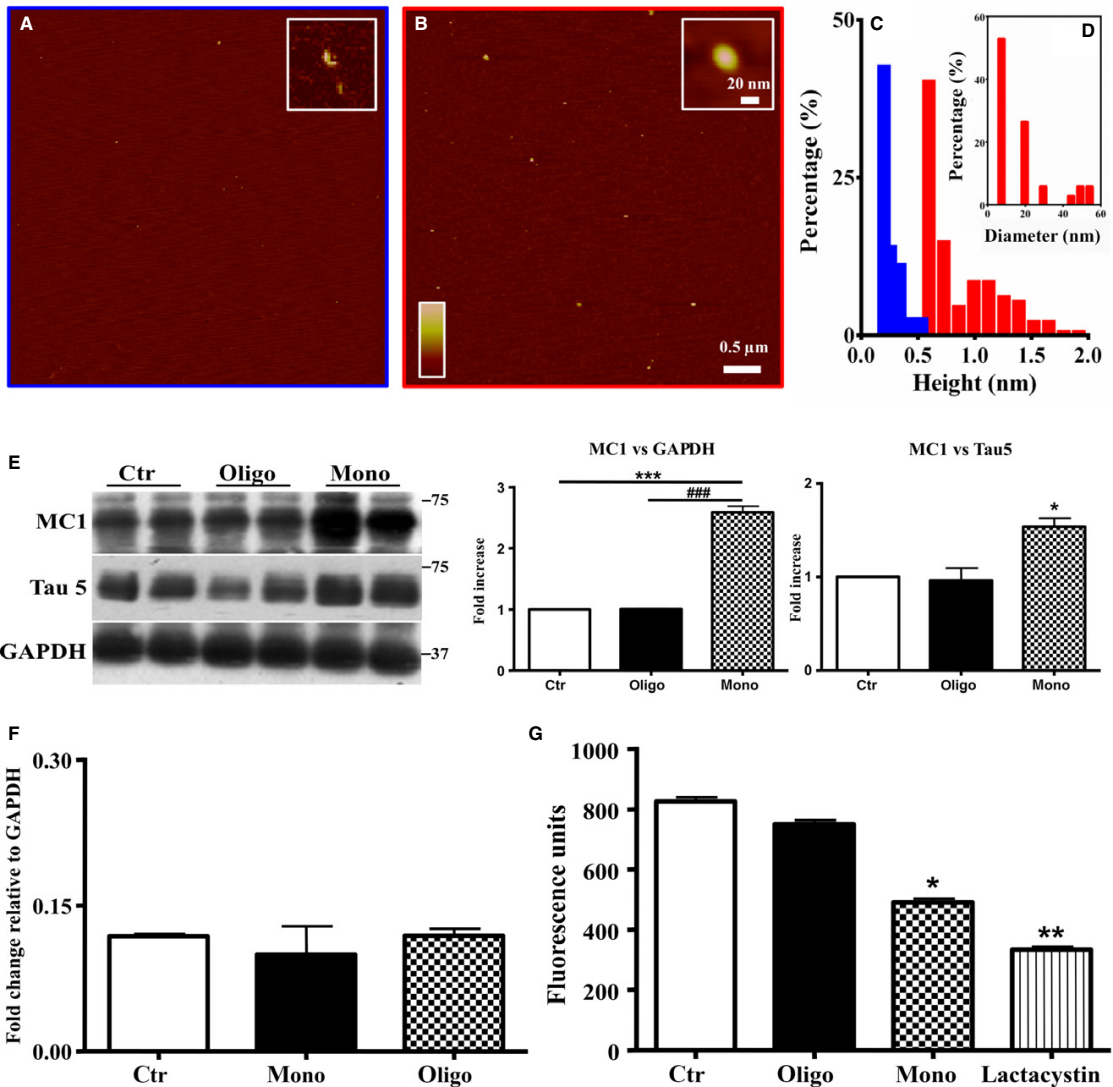
Aβ1‐42 monomers induce a conformational change of Tau protein. (A‐B) Analysis of the molecular assemblies of monomeric and oligomeric forms of Aβ 1‐42. (C) Representative atomic force microscopy (AFM) images of monomeric (initial state) and oligomeric assemblies, respectively. (D) Histogram representation of the heights of monomeric (blue bars) and oligomeric (red bars) assemblies, respectively. Distribution of the diameter histogram of oligomeric assemblies. (E) Representative Western blot of brain extracts from control (saline) and treated (Aβ1‐42 peptides by ICV for 3 h) mice (2‐month‐old) using a conformational Tau antibody (MC1) and a total Tau antibody (Tau5) for detection. An antibody raised against GAPDH or Tau 5 served as loading control. Densitometric quantification shows an increase of the total protein level of both MC1 and Tau5 induced by monomers. (F) Quantitative real‐time PCR (qPCR) analysis on total RNA extracted from brains of control and treated mice. Both treatments were not able to affect gene transcription. GAPDH mRNA expression was used as the internal standard. (G) Proteasomal activity assay on brain extracts from control and treated mice. Monomers halved the proteasomal activity. Lactacystin was used as a positive control. (H) Representative Western blot of brain extracts from control (saline) and treated with Aβ1‐42 monomeric as well as oligomeric depsipeptides by ICV for 3 h) mice (2‐month‐old) using a conformational Tau antibody (MC1). An antibody raised against GAPDH served as loading control. Densitometric quantification shows an increase of the total protein level of both MC1 induced by depsi‐monomers. The data are mean ± standard error of the mean (SEM), *P < 0.05; **P < 0.01 vs. control by one‐way ANOVA followed by Bonferroni *post hoc* test, *n* = 6.

We performed all experiments injecting 1 μL Aβ1‐42, monomers and oligomers, 0.2 μm, in 2‐month‐old hTau mice, sacrificed after 3 h and 4 days. The results are the average of four identical experiments. The two preparations were controlled with atomic force microscopy. Peptide was analyzed immediately after switching and after incubation for 24 h at 4 °C. Scanning Probe Image Processor (spip) software was used to analyze the distribution of species in terms of heights (Fig. 1C) and diameters (Fig. 1D). This software enables the elaboration of atomic force microscopy (AFM) images, and it specifically takes into account the features of the tips and the tapping mode. Therefore, it is able to obtain very accurate data on the height and diameter of the molecular assemblies formed by Aβ peptides (Munz, 2013). Figure 1A,B show AFM images obtained at the initial or oligomeric state of Aβ1‐42, respectively. The analysis of the sample at the initial state showed a presence of assemblies, ranging from 0.2 to 0.5 nm in height (Fig. 1C, blue bars). The incubation for 24 h at 4 °C leads to the presence of oligomers with a more broad distribution in height ranging from 0.5 to 2.0 nm (Fig. 1C, red bars) and the appearance of two families in the range 10–25 nm (85%) and 40–60 nm (15%) in diameter (Fig. 1D).

### Aβ1‐42 monomers, but not oligomers, induce conformational change of Tau protein

To determine whether Aβ1‐42 monomers and oligomers affect Tau conformation, we performed Western blot analysis using the antibody MC1. MC1 is a conformation‐dependent antibody that recognizes an early pathological Tau conformation (Weaver *et al*., 2000). As showed in Fig. 1E, 0.2 μm Aβ1‐42 monomers after 3 h induced Tau protein conformational change as revealed by the significant increase of the corresponding band (3.5‐fold increase). Monomers are also able to mediate a significant (twofold) increase of total Tau (Fig. 1E). Oligomeric forms of Aβ1‐42 do not modify these parameters (Fig. 1E). We normalized the levels of bands obtained with MC1 antibody not only with GAPDH but also with total Tau levels. We found that the conformational change of Tau is significant after normalization of the bands with GADPH antibody (2.5‐fold) and remains slightly significant after normalization with total Tau (1.6‐fold). Because the conformational change recognized by MC1 is found in PHF, but not in normal brain, it has been suggested that the formation of the MC1 epitope is one of the earliest pathological alterations of Tau in AD (Weaver *et al*., 2000). For this reason, we presumed that the effect mediated by Aβ142 monomers on conformation of Tau is not invalidated by the increase of total Tau. Next, we investigated whether the increase of total Tau, shown after treatment with monomers, was due to increased expression of the protein or to inhibition of its degradation. We found that the injection of mice with both preparations of Aβ1‐42 did not increase mRNA levels of Tau, evaluated with quantitative real‐time PCR (qPCR) (Fig. 1F). Thus, we evaluated whether monomeric preparations were able to alter the proteasomal degradation of Tau, thus increasing its concentration in free state. As reported in Fig. 1G, the injection with monomers of Aβ1‐42 halved the proteasomal activity, in comparison with control animals as well as to mice injected with oligomers. As positive control, we used lactacystin, a streptomyces metabolite, which inhibits the proteasomal degradation of proteins targeting the 20S proteasome (Koh *et al*., 2005), to test the validity of the kit. As shown, lactacystin decreased proteasomal activity by approximately 70%.

For a further confirmation of the results, we used 1 μL of 0.2 μm of ‘depsi’ Aβ1‐42. This is a new synthetic strategy (‘depsipeptide’ technique) that allows to obtain reliable seed‐free solutions of monomers as well as oligomers of Aβ1‐42 (Beeg *et al*., 2011; Stravalaci *et al*., 2011). Again, the monomers represent the only preparation capable of inducing conformational change of the Tau protein, as revealed by MC1 conformational antibody (Fig. 1H).

**Figure 2:**
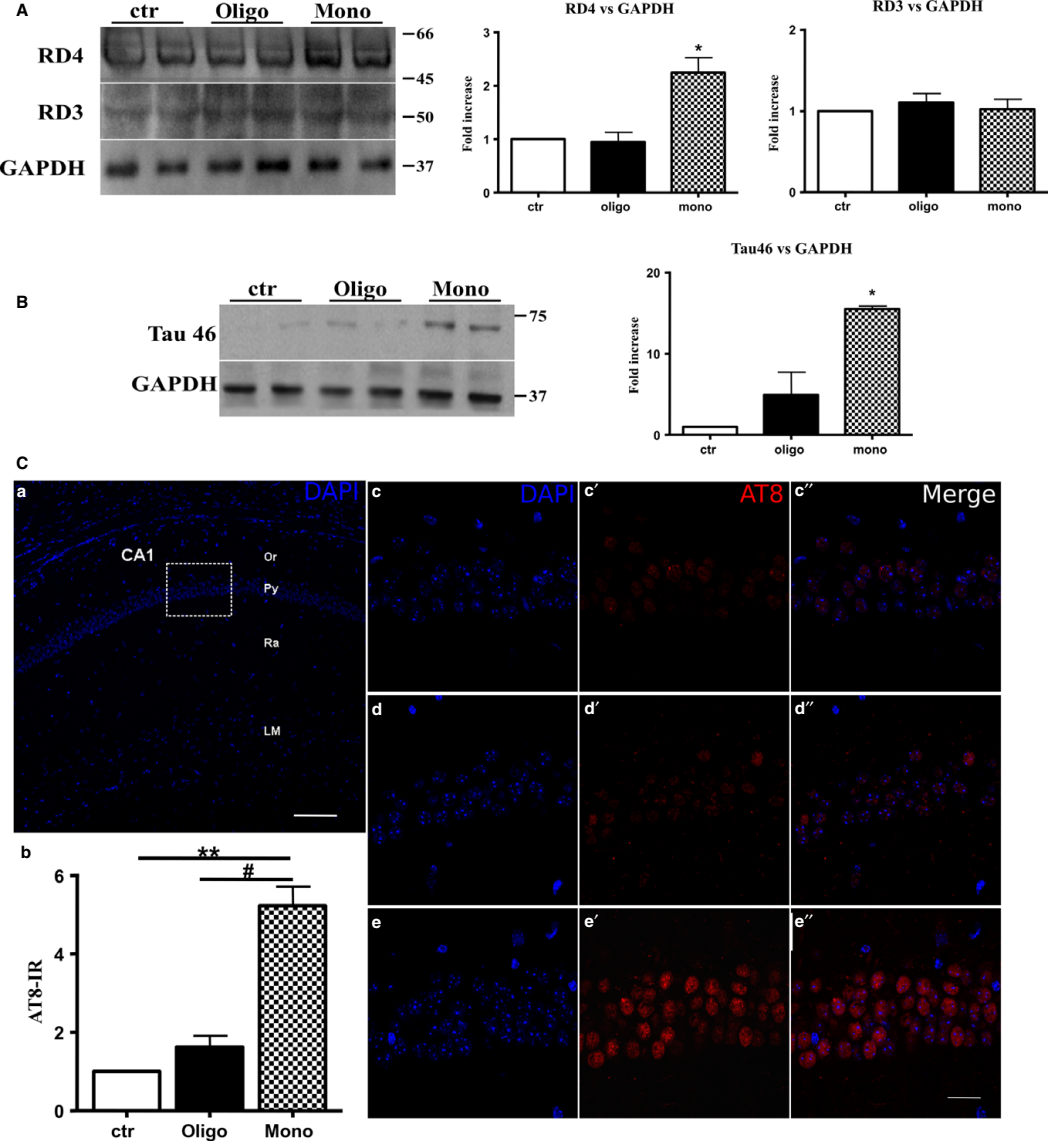
Aβ1‐42 monomers produce alternative splicing, insoluble Tau aggregates, and hyperphosphorylation of Tau protein. (A) Representative Western blot of brain extracts from control (saline) and treated (Aβ1‐42 peptides by ICV for 3 h) mice (2‐month‐old) using a 4‐repeat Tau isoform RD4 and 3‐repeat Tau isoform RD3 for detection. An antibody raised against GAPDH served as loading control. Densitometric quantification shows an increase of the isoform with four binding domains mediated by monomers. (B) Representative Western blot of insoluble Tau fraction by sarkosyl detergent technique extracts from control (saline) and treated (Aβ1‐42 peptides by ICV for 3 h) mice (2‐month‐old) using a Tau 46 antibody for detection. An antibody raised against GAPDH served as loading control. Only after treatment with monomeric preparations was present a band at approximately 75 kDa molecular weight. (C) a. Schematic image of the mouse hippocampus CA1 region (coronal section). b. Quantification of AT8‐immunoreactive (IR) cells by imagej NIH software. The data are mean ± standard error of the mean (SEM),**P < 0.01 vs. ctr and #*P* < 0.05 vs. oligo by one‐way ANOVA followed by Bonferroni *post hoc* test; *n* = 9. c–e″. Higher magnifications of the box area in a. showing pyramidal layer of control and treated mice (Aβ1‐42 peptides by ICV for 3 h) stained with an antibody that recognizes PHF‐Tau (AT8 in red). Nuclei are counterstained with DAPI (blue). Scale bars: 100 μm in a, 20 μm in c–e″.

Then, we better characterized the conformational change mediated by Aβ1‐42 preparations on Tau protein. First, we investigated whether Aβ1‐42 preparations changed the levels of three‐repeat (RD3) and/or four‐repeat (RD4) Tau in hTau mice. We showed that monomeric preparations of Aβ1‐42 were able to slightly but significantly (twofold) increase the levels of 4R Tau over 3R Tau (Fig. 2A). Oligomers did not affect these parameters (Fig. 2A).

Then, we studied the insoluble Tau fraction by sarkosyl detergent technique, and we showed that only after treatment with monomeric preparations was present a band at approximately 75 kDa molecular weight revealed by Tau 46 antibody (Fig. 2B).

### Aβ1‐42 monomers induce hyperphosphorylation of Tau protein

To determine whether Aβ1‐42 monomers and oligomers modify phosphorylation of Tau protein, we measured levels of phosphorylation using AT8 antibody that recognizes Ser202/Thr205 phospho‐epitopes.

We performed a pilot experiment to evaluate, by fluorescence, whether our treatment (Aβ1‐42 peptides by ICV for 3 h) affected Tau phosphorylation. Sections from hippocampus were immunostained with Tau monoclonal antibody AT8. We observed immunoreactivity in the perinuclear region, primarily in the CA1 pyramidal layer (Fig. 2 Cc′–c″, d′–d″, e′–e″). Phospho‐Tau reactivity was evaluated by imagej software and resulted statistically significant in animals injected with monomeric preparations (Fig. 2 Cb) compared to those treated with oligomers or control (one‐way ANOVA, *P* < 0.05 vs. oligo, *P* < 0.01 vs. ctr) (Fig. 2Cb, d′–d″, e′–e″).

**Figure 3:**
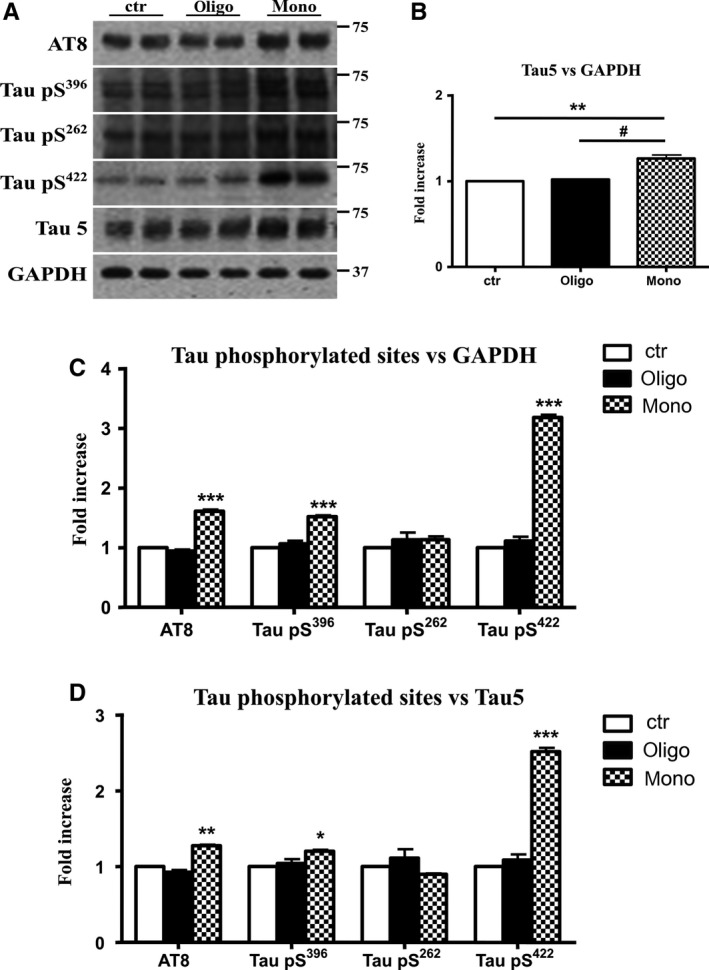
Aβ1‐42 monomers promote phosphorylation at particular sites that have been related to Alzheimer's disease (AD) progression. Representative Western blot of brain extracts (20 μg protein) from control (saline) and treated (Aβ1‐42 peptides by ICV for 3 h) mice using antibodies specific for the detection four pathological Tau phosphorylation sites: AT8, pS396, pS262, and pS422. An antibody raised against GAPDH or Tau 5 served as loading control. Densitometric quantification shows an increase of the total protein level of AT8, pS396, and pS422 induced by monomers while monomeric and oligomeric preparations did not change pS262 expression. The data are mean ± standard error of the mean (SEM), *P < 0.05; ***P < 0.001 vs. control by one‐way ANOVA followed by Bonferroni *post hoc* test, *n* = 6.

To confirm the results obtained with immunofluorescence, we measured the levels of hyperphosphorylated Tau using AT8 antibody in Western blot. Aβ1‐42 monomers significantly (1.8‐fold) increased hyperphosphorylation of Tau, whereas oligomers did not modify the parameter (Fig. 3). We confirmed this result normalizing the amount of phosphorylation with the levels of total Tau, revealed by antibody Tau5, and we found that the increase of Tau phosphorylation remains slightly significant also after normalization with total Tau (Fig. 3). Whether Tau hyperphosphorylation in AD is a cause of aggregation (Alonso *et al*., 2001) or whether the two changes occur independently is still controversial. We tested the ability of Aβ1‐42 monomers and oligomers to promote phosphorylation at other particular sites that have been related to AD progression. Thus, we determined the residues affected by the injection of Aβ1‐42 monomers and oligomers using phosphorylation‐sensitive antibodies, such as Tau S396, S422, and S262. As reported in Fig. 3, the oligomers of Aβ1‐42 did not induce phosphorylation in the studied sites. Monomers determined a 1.5‐fold and 1.3‐fold increase in phosphorylation of Tau at S396 respective to GADPH or Tau 5 normalization (Fig. 3); the residue S262 was not hyperphosphorylated if normalized on GADPH or total Tau levels (Fig. 3). The lack of phosphorylation at this site is not in agreement with literature data reporting that levels of Ser262 phosphorylation were increased by Aβ1‐42, but in those studies were not used Aβ monomers (Qureshi *et al*., 2013; Ando *et al*., 2016). The more significant data are the 3.5‐fold and 2.8‐fold increase of Tau phosphorylation at position S422 with respect to GAPH or Tau normalization (Fig. 3).

**Figure 4:**
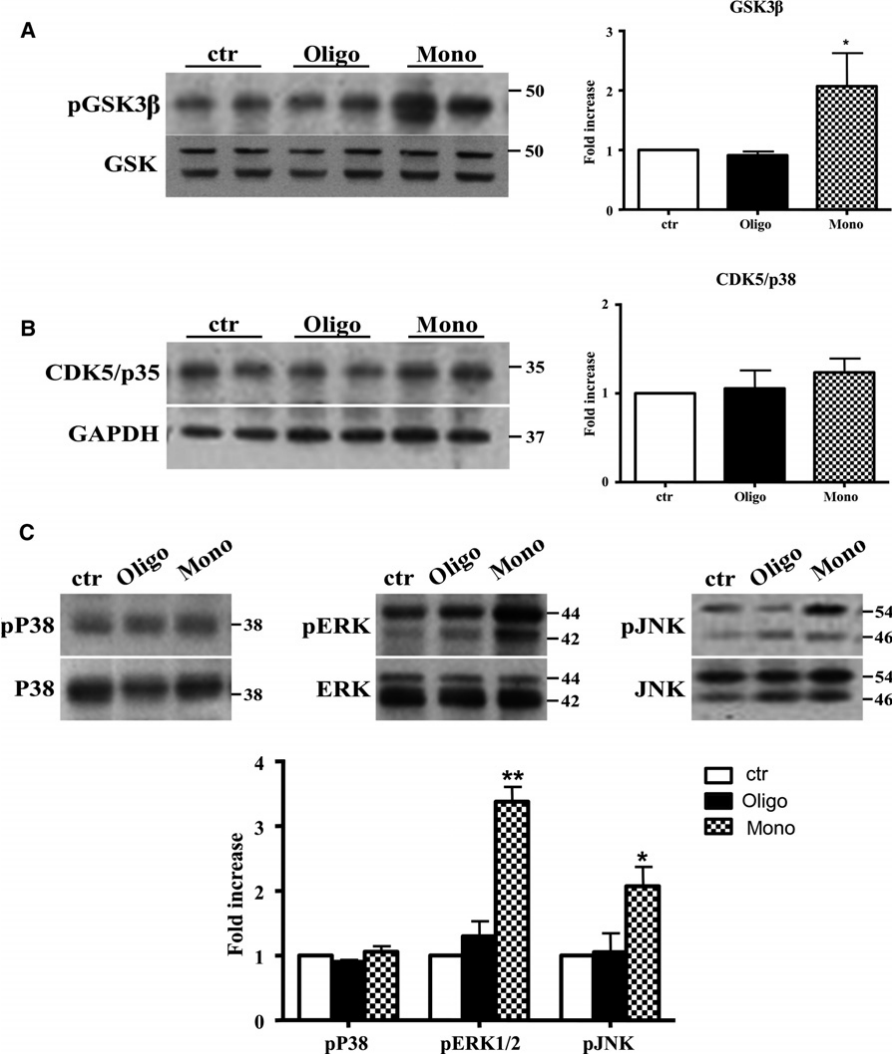
Aβ1‐42 monomers affect Tau phosphorylation through GSK3β, ERK1/2, and JNK kinases activation. Representative Western blot of brain extracts from control (saline) and treated mice using pGSK3β (A), CDK5/p35 (B), pP38, pERK1/2, and pJNK (C) antibodies for detection. Densitometric quantification shows an increase in the total protein level of pGSK3β induced by monomers (A), while CDK5/p35 was not modified by any treatments (B). (A,B) An antibody raised against GAPDH was used as loading control. The data are mean ± standard error of the mean (SEM), *P < 0.05 vs. control by one‐way ANOVA followed by Bonferroni *post hoc* test, *n* = 6 for each kinase. (C) pP38, pERK1/2, and pJNK levels were standardized against their respective total protein amount. Densitometric quantification shows an increase of ERK1/2 and JNK activity due to monomers, while p38 was not involved by any treatments. The data are mean ± standard error of the mean (SEM), *P < 0.05; **P < 0.01 vs. control by two‐way ANOVA followed by Bonferroni *post hoc* test, *n* = 3 for each kinase.

Previous studies reported that Tau is phosphorylated at the aforementioned sites by phospho‐kinases such as GSK3β, mitogen‐activated protein kinase (MAPK), and cyclin‐dependent kinase (CDK)5 (Fontaine *et al*., 2015). Thus, we tested the increase in the concentration of these kinases in our experimental model 3 h after injection of monomers and oligomers of Aβ1‐42 in hTau mice. First, we studied GSK3β, the major protein kinase regulating Tau phosphorylation in the brain (Hernandez *et al*., 2013). We found that injection of Aβ1‐42 monomers was followed by an increase (twofold) of pGSK3β protein levels (Fig. 4A) Then, we tested the levels of CDK5 a protein serine/threonine kinase (Wilkaniec *et al*., 2016). As reported in Fig. 4B, levels of CDK5/p35 were unmodified by injection of monomeric preparations; thus, in our experimental model, this kinase does not seem involved in the conformational change of Tau. Finally, we measured levels of MAPK such as p38, ERK1/2, and JNK. As reported in Fig. 4C, injection of hTau mice with monomeric preparations was followed by an activation of the JNK pathway, as shown by significant increase (twofold) in levels of phospho‐JNK. In addition, monomers of Aβ1‐42 also induced a significant increase (3.5‐fold) of pERK1/2 protein levels without affecting p38 pathway (Fig. 4C). Oligomers did not activate the three kinases (Fig. 4A–C).

**Figure 5:**
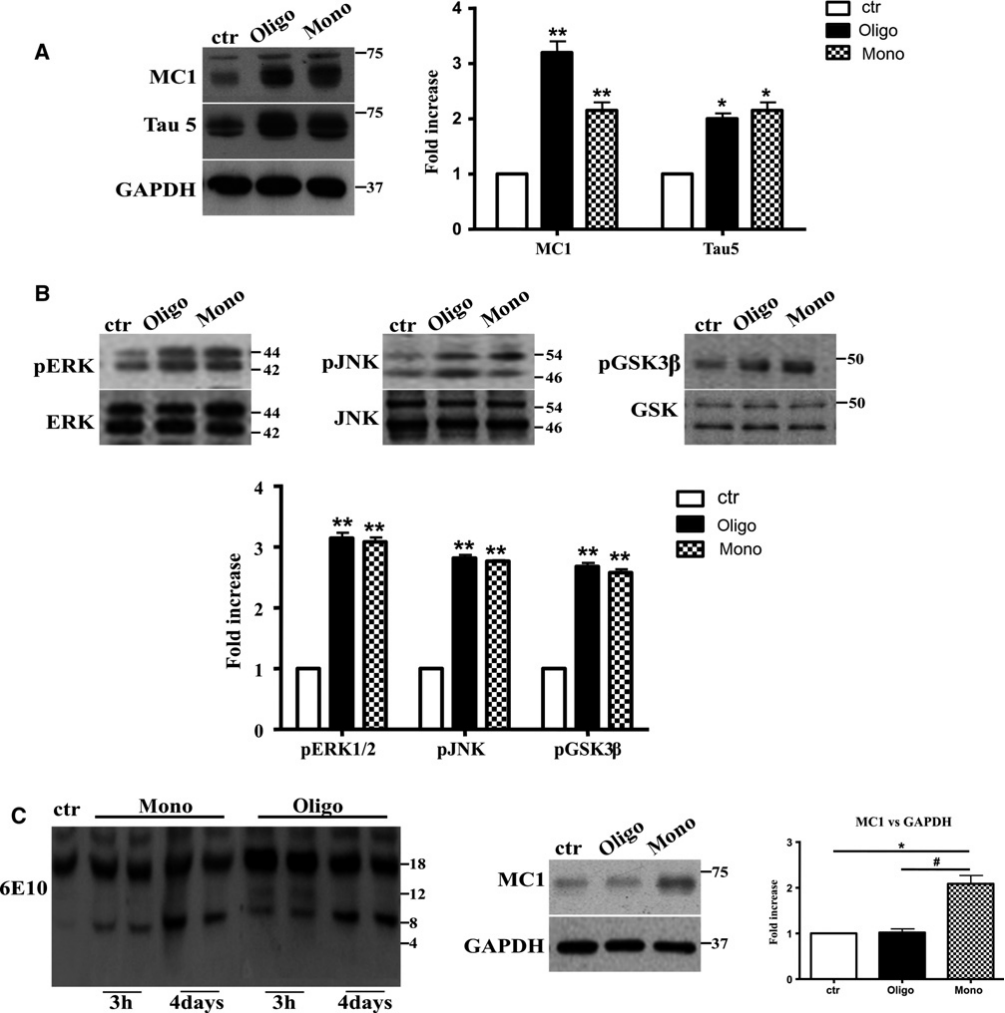
After 4 days of treatment, Aβ1‐42 oligomers and the monomers show a similar effect on Tau protein and phospho‐kinases. Representative Western blot of brain extracts from control (saline) and treated mice using MC1 and Tau5 (A), pERK1/2, pJNK, and pGSK3β (B) antibodies for detection. (A) Densitometric quantification shows an increase in the total protein level of both MC1 and Tau5 induced by both preparations. An antibody raised against GAPDH served as loading control. The data are mean ± standard error of the mean (SEM), *P < 0.05, **P < 0.01 vs. control by one‐way ANOVA followed by Bonferroni *post hoc* test, *n* = 3. (B) Densitometric quantification shows an increase of ERK1/2 and JNK activity due to both monomers and oligomers treatments. pERK1/2 and pJNK levels were standardized against their respective total protein amount. The data are mean ± SEM, **P < 0.01 vs. control by two‐way ANOVA followed by Bonferroni *post hoc* test, *n* = 3 for each kinase. (C) Representative Western blot of the aggregation state of Aβ1‐42 in brain tissue of hTau mice injected after 3 h or 4 days with both preparations using the 6E10 antibody. As shown, the aggregation state of oligomeric preparation was different in the short and in the longer treatment: After 3 h, a band of approximately 12 Kda was observed, whereas after 4 days, this band was no more detectable. The data are mean ± SEM, **P < 0.01 vs. control by two‐way ANOVA followed by Bonferroni *post hoc* test, *n* = 3.

The mechanisms by which the self‐assembly of Aβ1‐42 leads to toxicity are not well understood as all intermediated aggregates are transient and in dynamic balance (Kaden *et al*., 2012). Thus, we studied whether a longer treatment of hTau mice, with both oligomeric as well as monomeric preparations of Aβ1‐42, would mediate different effects on Tau. Mice were injected ICV with 1 μL 0.2 μm monomeric as well as oligomeric Aβ1‐42 and sacrificed 4 days later. We found that the longer treatment with oligomers induced effects that mimic those observed with monomers. As reported in Fig. 5A, we found that oligomers significantly increase (threefold) the aberrant conformation of Tau as revealed by MC1 conformational antibody, as well as the amount of total Tau (twofold increase). Monomers maintain the ability to modify the conformation and the amount of Tau at the same levels of the shorter treatment. We also tested the increase in concentration of GSK3β, pERK1/2, and pJNK to confirm the ability of oligomers to modify the signal pathways activated after 3 h treatment with monomers of Aβ1‐42.

As expected, after 4 days of treatment, oligomeric preparation was able, as much as monomers, to activate both MAPK kinases, ERK1/2 and JNK, and GSK3β pathways as shown by the significant increase (approx. threefold) in levels of phosphorylation of ERK 1/2, JNK, and pGSK3β (Fig. 5B). The different effect of oligomeric preparation on Tau conformation after longer treatment could be ascribed to their poor diffusibility in the brain after 3 h. But, it has been reported that large oligomers of Aβ1‐42 after 6 h are clearly able to enter N2A cells (Manzoni *et al*., 2011). An alternative hypothesis could be that after 4 days, Aβ oligomers break into smaller aggregates. We tested this hypothesis by immunoblotting brain tissue of hTau mice injected after 3 h or 4 days with both preparations (Fig. 5C) with the 6E10 antibody, specific for the 1‐16 residues of Aβ. As shown, in tissues of mice injected with monomers after 3 h or 4 days, a band of approx. 8 kDa was observed. The aggregation state of oligomeric preparation was different in the short and longer treatments: After 3 h, two bands of approximately 12 and 8 kDA were observed, whereas after 4 days, the higher band was no longer detectable, and the aggregation state became almost completely similar to the monomeric form (Fig. 5C). We cannot observe with this experiment a band of 4 kDa (monomers), because the amount of beta‐amyloid administered is well below the sensitivity of the method.

### The phosphorylation of Tau mediated by Aβ1‐42 monomers is crucial to mediate the conformational change

**Figure 6:**
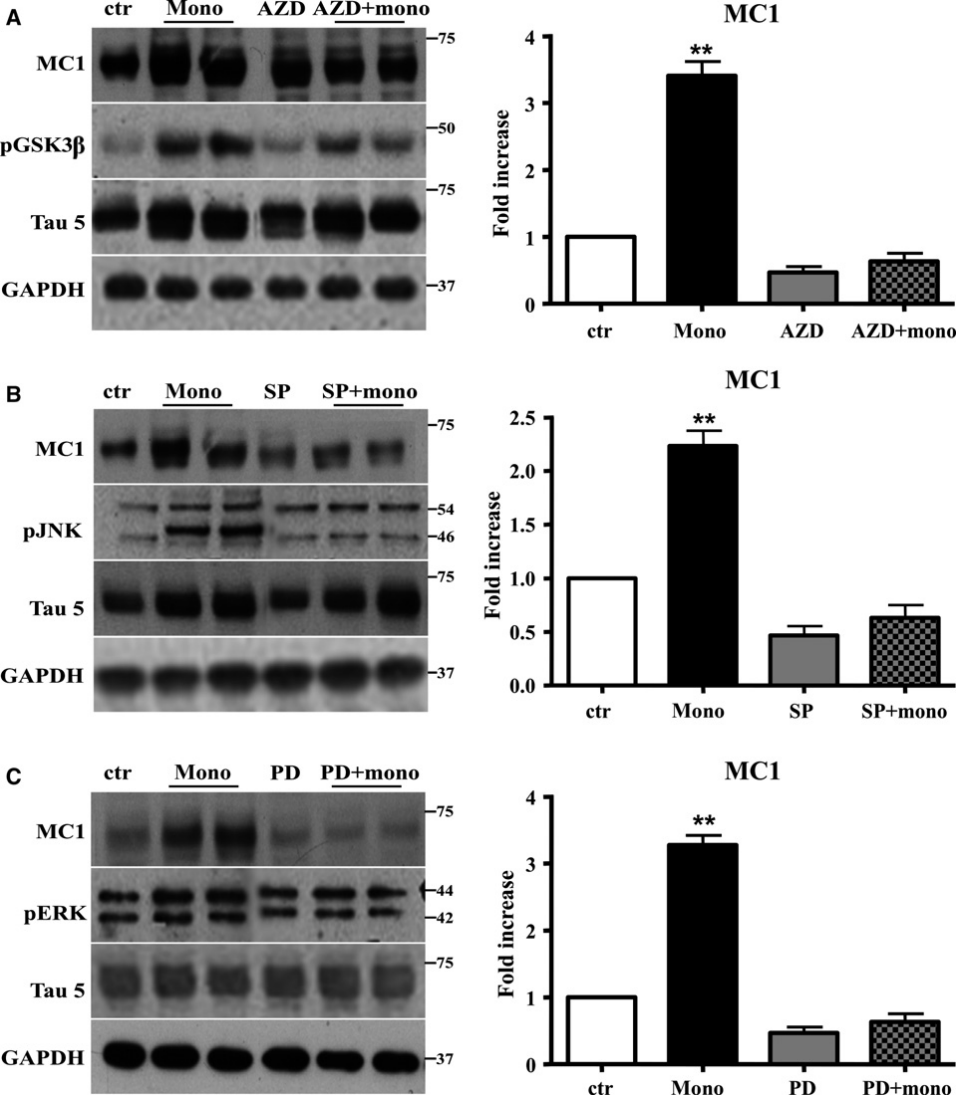
The activation of JNK, ERK1/2, and GSK is required to mediate the conformational change of Tau protein induced by Aβ1‐42 monomers. Representative Western blot of brain extracts from control and pretreated or not with the GSK3β inhibitor AZD1080, ERK inhibitor PD98059, and JNK inhibitor SP600125 before 3 h injection with Aβ1‐42 Tau mice using MC1 (A‐C), pGSK3β (A), pJNK (B), and pERK1/2 (C) antibodies for detection. An antibody raised against GAPDH or Tau5 served as loading control. Densitometric quantification shows that each pretreatment was followed by a complete inhibition of the pathway and by the complete reversion of Tau conformational change (A–C). The data are mean ± standard error of the mean (SEM), **P < 0.01 vs. control by one‐way ANOVA followed by Bonferroni *post hoc* test, *n* = 6 for each kinase.

Finally, we studied whether an increase in concentration of phospho‐kinases was needed for the conformational change of Tau induced by Aβ1‐42 monomers. To investigate whether GSK3β was involved in conformational change and aggregation of Tau, the GSK3β inhibitor AZD1080 was given via oral gavage 6 h before the injection of mice with monomeric preparation of Aβ1‐42. As reported in Fig. 6A, the pretreatment with GSK3β inhibitor was able to rescue the increase of the pathway and to reverse the conformational change of Tau.

To inhibit MAPK, ERK1/2, and JNK, we used PD98059 and SP600125, respectively, injected ICV 30 min before monomeric preparation of Aβ1‐42. Also in this case, we found that the pretreatment was followed by a complete inhibition of the pathways and by the complete reversion of Tau conformational change (Fig. 6B,C).

## Discussion

This study's purpose was to investigate whether and how Aβ1‐42 modifies the conformation of Tau to render it prone to aggregate, in a PHF‐like state. We used a mouse model that expresses wild‐type human Tau and does not spontaneously produce Tau aggregates. In previous reports, the capability of Aβ1‐42 to induce Tau aggregation was studied in mice expressing mutant human Tau, models of tauopathies (Lewis *et al*., 2001; Oddo *et al*., 2006; Rhein *et al*., 2009; Nisbet *et al*., 2015).

We tested the effect on Tau of either oligomers or monomers of Aβ1‐42. Aβ oligomers lower neuronal activity, through various mechanisms (Bero *et al*., 2011; Brouillette *et al*., 2012; Pitt *et al*., 2013; Tu *et al*., 2014; Xu *et al*., 2014; Lazzari *et al*., 2015). Instead, Aβ monomers enhance various cellular functions (Giuffrida *et al*., 2010). We previously showed that they increase receptor for advanced glycation end products (RAGE) expression (Piras *et al*., 2014), BACE1 protein levels (Piccini *et al*., 2012), and inhibit lysosomal activity, through the activation of the nuclear factor (NF)‐κB pathway (Guglielmotto *et al*., 2012). Therefore, Aβ oligomers and monomers have completely different pathologic and physiologic effects (Guglielmotto *et al*., 2014).

Our experiments show that Aβ monomers, but not oligomers, after 3 h of intraventricular injection in mice expressing wild‐type human Tau, change Tau conformation, displaying an epitope present only in pathological Tau aggregates (Weaver *et al*., 2000). In the same experimental conditions, Aβ monomers induce two phosphorylated epitopes not present in normal Tau (Ser396 and Ser422) and activate GSK3β and MAPK, two kinases responsible for phosphorylation at these two residues. Indeed, inhibition of the kinases rescues phosphorylation of Tau. Moreover, inhibition of kinases hampers the conformational change of Tau determined by Aβ monomers.

Then, we asked whether the observed modifications of Tau could depend on an increase of Tau expression and/or protein levels. The increase of total Tau leads to higher levels of its protein levels, a condition that favors phosphorylation and aggregation, as demonstrated with mutant Tau, when unable to bind tubulin (Holton *et al*., 2001; Bunker *et al*., 2006). We observed that Aβ monomers, in parallel with an increase of Tau protein levels, impair proteasomal degradation. Instead, Tau mRNA is unchanged. Thus, Aβ monomers alter Tau conformation through two mechanisms: hyperphosphorylation and increase of total Tau levels. Their relative influence in producing Tau aggregation remains to be determined. Moreover, the results show that Aβ does not have a direct nucleation effect on Tau.

Aβ1‐42 oligomers recapitulate all the described alterations of Tau only after 4 days from the intraventricular injections. At this time, oligomers have the same electrophoretic pattern of monomers in mice brains, as shown in Fig. 6C. It is known that Aβ monomers, oligomers, and fibrils are in dynamic balance in AD brains (Nisbet *et al*., 2015).

Aβ1‐42 is certainly implicated in normal neuronal activity. Its concentration in interstitial fluid is proportional to the level of global brain function (Fagan *et al*., 2009). Picomolar concentrations of Aβ1‐42 foster synaptic activity (Puzzo *et al*., 2008). Higher Aβ1‐42 levels have the opposite effect (Puzzo & Arancio, 2013). It is reasonable to speculate that low, physiologic, and higher, pathologic, concentrations of Aβ1‐42 correspond to monomers and oligomers of the peptide, respectively.

With this background, we propose that Aβ1‐42 has normally the role of promoting neuronal plasticity, enhancing the levels of Tau and accelerating the cellular signaling that favors dendritic sprouting. The exaggeration of these effects, determined by increase of the levels of Aβ monomers, causes Tau aggregation, finally leading to neurofibrillary pathology.

Our results have practical implications; currently, the major efforts of Alzheimer's disease therapy are focused on removal of Aβ oligomers, and not monomers.

## Experimental procedures

### Mice and ICV

hTau mice (Mapt ^tm1(EGFP)Klt^Tg(MAPT) 8cPdav/J; #004808, Jackson Laboratory) were crossed with Tau knockout (KO) mice (Mapt ^tm1(EGFP)Klt^/J; #004779, Jackson Laboratory, Bar Harbor, ME USA), to obtain pregnant females carrying hTau fetuses as described by Andorfer *et al*. (2003). Mice were genotyped by PCR assay using the following primers: human tau transgene (forward 5′‐ACTTTGAACCAGGATGGCTGAGCCC‐3′, reverse 5′‐CTGTGCATGGCTGTCCCTACCTT‐3′), mouse tau gene (forward 5′‐CTCAGCATCCCACCTGTAAC‐3′, reverse 5′‐CCAGTTGTGTATGTCCACCC‐3′), and disrupted tau gene (forward 5′‐CAGGCTTTGAACCAGTATGG‐3′, reverse 5′‐ TGAACTTGTGGC CGTTTACG‐3′). Mice were maintained on a Swiss Webster/129/SvJae/C57BL/6 background (Andorfer *et al*., 2003) (See Fig. S1 in Supporting Information).

Animals were kept on a 12‐h light/dark cycle with food and water available *ad libitum*. All experimental procedures on live animals were performed under the supervision of a licensed veterinarian, according to: (i) European Communities Council Directive (November 24, 1986; 86/609/EEC), (ii) Italian Ministry of Health and University of Torino's institutional guidelines on animal welfare (DL 116/92 on Care and Protection of living animals undergoing experimental or other scientific procedures; authorization No. 17/2010‐B, June 30, 2010), and (iii) *ad hoc* Ethical Committee of the University of Turin (http://www.unito.it/ricerca/strutture-la-ricerca/comitato-di-bioetica-dellateneo).

Two groups of 2‐month‐old male mice were used: (i) treated for 3 h (*n* = 60) and (ii) treated for 4 days (*n* = 25). Under isoflurane O_2_/N_2_O anesthesia, hTau mice (*n* = 100) were ICV injected with Aβ peptides or saline. Coordinates used for injection were anteroposterior, −0.5 mm; lateral, 1.2 mm relative to bregma and dorsoventral, 1.7 mm from the dural surface. The method was validated by injecting one mouse with trypan blue (1 μL).

### Treatments

Mice were injected with 0.2 μm Aβ1‐42 peptides (#20276, Anaspec). The lyophilized synthetic peptides were dissolved in 1.0% of NH_4_OH to obtain a clear solution and stored at −20 °C in aliquots. Monomeric preparations were brought to 0.2 μm (final concentration) with sterile double‐distilled water, centrifuged at 10 000 ***g*** for 10 min to remove possibly aggregate and immediately added to the cell culture. Oligomeric preparations were maintained at 4 °C for 24 h and then injected.

The quality of Aβ preparations was controlled using AFM. AFM was carried out on a Multimode AFM with a Nanoscope V system operating in tapping mode using standard antimony(n)‐doped Si probes (*T*: 3.5–4.5 mm, *L*: 115–135 mm, *W*: 30–40 mm, f0: 313–370 kHz, *k*: 20–80 N m^−1^) (Bruker). The scan rate was tuned proportionally to the area scanned and was kept in the 0.5–1.2 Hz range. The sample was then diluted to 5 μm with PBS, and 50 μL of solution was spotted onto a freshly cleaved muscovite mica disk and incubated for 5 min. The disk was then washed with ddH_2_O and dried under a gentle nitrogen stream. Samples were analyzed with the spip (version 5.1.6 released April 13, 2011) data analysis package (Nanoscience Instruments, Phoenix, AZ, USA). spip software was used to analyze the distribution of the molecular assemblies of the different populations in terms of height and diameter, as previously described (Messa *et al*., 2014).

*DEPSI‐*Aβ1–42 peptide was synthesized using depsipeptide method as previously described (Beeg *et al*., 2011; Stravalaci *et al*., 2011). Aβ1–42WT was stored in water: trifluoroacetic acid, 0.02% at a concentration of 150 μm. The depsipeptide method, through the introduction of *O*‐acyl isopeptide structure into the Gly‐25–Ser‐26 sequence stable at acidic pH, is able to inhibit the self‐aggregation of the peptide. Upon a change to basic pH (switching procedure), the depsipeptide is converted to the Aβ1–42 native sequence.

After the switching, Aβ1–42 solution was brought to a final concentration of 100 μm in 50 mm phosphate buffer, pH 7.4, and incubated for 24 h at 4 °C to obtain the oligomer rich solution (Beeg *et al*., 2011; Stravalaci *et al*., 2011).

The number of mice/group was determined with a standard power analysis (Faul *et al*., 2007). We set the significance level (probability of a type‐I error) at 5% (α = 0.05) and chose a sample size that would minimize the probability of type‐II errors (β ≤ 10%). From our initial experiment, we obtained estimates of the expected effects of the 3 h ICV treatment, and variability for a given number of mice determining that a sample size of nine animals in each treatment group would be sufficient to detect a 30% difference in immunoreactivities (described below) with a statistical power of 95%. Our controls were hTau mice ICV injected with saline or with Aβ1‐42 scramble (Anaspec S‐25382). Intraventricularly, injection of hTau mice with scramble peptide did not modify the conformational change on Tau with respect to saline control (see Fig. S2 in Supporting Information). To investigate whether c‐Jun N‐terminal kinase (JNK), extracellular signal regulated MAP kinase (ERK)1/2, and glycogen synthase kinase (GSK)3β are involved in Aβ1‐42‐induced conformational change of Tau, we used three different groups of 2‐month‐old animals killed 3 h after the Aβ1‐42 injection, one for inhibitor. The peptides JNK inhibitor SP600125 (5 μg μL^−1^) (#20276; Santa Cruz Biotechnology, Dallas, Texas, USA) and ERK inhibitor PD98059 (0.1 mg mL^−1^) (#20276, Santa Cruz Biotechnology) were injected ICV 30 min before Aβ1‐42 injection. The peptide GSK3 inhibitor AZD1080 (1 μm mL^−1^, #S7145; Selleckchem, Houston, TX, USA) was given via oral gavage 6 h before Aβ1‐42 injection.

### Antibodies and immunoblot analysis

Immunoblot analysis was performed using the following antibodies: MC1 (kind gift from Dr. P Davies, Albert Einstein College of Medicine, New York, 1:500); Tau5 (Millipore, #577801, 1:500); AT8 (Innogenetics, Alpharetta, GA, USA, #90206, 1:500); Tau 46 (Abcam, Cambridge, UK, #22261, 1:1000), Tau RD4 (Millipore, Billerica, MA, USA, #05‐804, 1:1000), Tau RD3 (Millipore #05‐803, 1:1000), TaupS396 (Invitrogen, #44752G, 1:1000); TaupS262 (Invitrogen, #44750G, 1:1000); TaupS244 (Invitrogen, Camerillo, CA, USA, #44764G, 1:1000); GSK3αpY279/βpY216 (Invitrogen, #44604G, 1:1000); GSK3α/β tot (1:1000, Invitrogen, #44610, 1:1000); GSK3βpS9 (Novex, Frederick, MD, USA, #710100, 1:1000); pJNK1/2 (Cell Signaling Technology, Beverly, MA, USA, #9251, 1:500); JNK1/2 (Cell Signaling Technology, #9252, 1:500); pP38 (Calbiochem, #506119, 1:1000); P38 (Calbiochem, San Diego, CA, USA, #06123, 1:1000); pERK1/2 (Cell Signaling Technology, #43765, 1:1000); ERK1/2 (Santa Cruz Biotechnology, Dallas, Texas, USA, Sc‐93, 1:1000); 6E10 (Chemicon, Billerica, MA, USA, Mab1560, 1:500); glyceraldehyde 3‐phosphate dehydrogenase (GAPDH) (Millipore, Mab374, 1:3000).

Fresh frozen brains were mechanically homogenized in ice‐cold buffer (25 mm Tris‐HCl pH7.4, 150 mm NaCl, 1 mm EGTA, 1 mm EDTA, 1 mm PMSF, phosphatase, and protease inhibitors) and then centrifuged at 10 000 ***g*** for 15 min al 4 °C to isolate soluble proteins. Supernatants (2 mg mL^−1^ solution) were collected and incubated with sarkosyl (1% final concentration) overnight at 4 °C. The sarkosyl mixtures were then centrifuged in Beckman SW 55 Ti rotor, Brea, CA, USA, at 116140 g for 1 h at 4 °C. Pellets were resuspended in 100 μL sample buffer to obtain sarkosyl‐insoluble proteins. Lysates (20 μg) were run on 3–8% Tris‐HCl gradient PAGE gel (Invitrogen) and then transferred to PVDF membrane. To determine the presence of Aβ1–42 oligomers in brain tissues, lysates were separated on 10–17.5% Tris–tricine gels, transferred onto nitrocellulose membranes. Blots were blocked (5% BSA) and incubated overnight at 4 °C with primary antibodies. Peroxidase‐conjugated secondary antibodies were incubated 1 h at room temperature (RT) and developed with Luminata Forte Western substrate (WBLUF0100, Millipore). Densitometric values were normalized to GAPDH.

### Immunofluorescence and microscopy

Brains were removed and cryoprotected in 30% sucrose after trans‐cardiac perfusion with 4% paraformaldehyde. Samples were cut into coronal free‐floating sections (25 μm). For immunofluorescence staining, sections were blocked and incubated overnight at 4 °C with AT8 (Thermo Fisher Scientific, Carlsbad, CA, USA, #MN1020, 1:25). Cy3‐conjugated secondary antibody (Jackson Immuno Research Laboratories, West Grove, PA, USA, 715‐165‐150, 1:200) was incubated 1 h at RT, and DAPI (Sigma Chemical Aldrich, Milwaukee, WI, USA) was used to stain nuclei. Controls included: Tau KO brains stained with AT8, and sections treated with secondary antibody alone. Neither showed appreciable staining. Images were acquired using Leica TCS SP5 confocal laser scanning microscopes (Leica, Richmond, IL, USA). The percentage of the overall AT8‐positive cells in the CA1 areas of hippocampus was quantified using the imagej NIH software for Windows (Bethesda, MA, USA).

### RNA extraction and quantitative real‐time PCR

Three hours after ICV injection, brain of 2‐month‐old male mice were homogenized in TRI‐Reagent (Sigma Chemical Aldrich) and total RNA was isolated. cDNA was synthesized using the M‐MLV Reverse Transcriptase (Invitrogen) and random primers. qPCR was performed using the qPCR Core kit for SYBR Green (Eurogentec, San Diego, CA, USA) on a StepOne real‐time PCR system (Life Technologies, Carlsbad, CA, USA). Samples were amplified simultaneously in triplicate in 1 assay run. Changes in mRNA levels were determined as the difference in threshold cycle (ΔCt) between the target gene and the reference gene. The following primers were used: 5′‐TGAACCAGGATGGCTGAGC‐3′ and 5′‐TTGTCATCGCTTCCAGTGC‐3′ for Tau exon2, 5′‐CCACCAACTGCTTAGCCCCC‐3′ and 5′‐GCAGTGATGGCATGGACTGTGG‐3′ for GADPH (internal standard).

### Proteasome activity assay

The proteasome activity assay was determined using a commercially available kit (Chemicon). The assay is based on detection of the fluorophore 7‐amino‐4‐methylcoumarin after cleavage from the labeled substrate LLVY‐AMC. The free AMC fluorescence can be quantified using a 380/460 nm filter set in a fluorometer.

### Statistical analysis

Statistical analyses were performed using graphpad prism version 4.0 (GraphPad Software, San Diego, CA, USA). All values were presented as mean ± standard error of the mean. Means were compared by one‐ or two‐way analysis of variance (ANOVA) with Bonferroni as a *post hoc* test. Values of *P < 0.05 were considered significant, **P < 0.01 very significant and ***P < 0.001 extremely significant.

## Conflict of interest

None declared.

## Author contributions

G.M. designed the study, performed the experiments, and analyzed the results; M.G.; R.B; RZ, LC collaborated in performing the experiments; G.P. analyzed the results and edited the manuscript; P.O. designed the study; O.A; MS designed the study; E.T. designed the study, analyzed the results, and wrote the manuscript; M.T. designed the study and wrote the manuscript.

## Supporting information


**Fig. S1** (A) Breeding scheme from mating hTau mice (Mapt ^tm1(EGFP)Klt^Tg(MAPT) 8cPdav/J; #004808, Jackson Laboratory); murine (m) Tau knock‐out (KO) mice (Mapt ^tm1(EGFP)Klt^/J; #004779, Jackson Laboratory) to generate hTau + mTauKO mice. (B, C) Specific PCR analysis of genomic DNA. Note: (B) transgene (tg) = 187 bp. (c) Mutant (KO) = 490 bp. 1Kb DNA ledder was used in both analysis

**Fig. S2** Representative western‐blot of brain extracts from control (saline) and mice injected with Aβ 1‐42 scramble preparation using MC1 (A) antibody for detection. Densitometric quantification did not reveal changes in the total protein level of MC1 induced by both treatments. An antibody raised against GAPDH served as loading control. *n* = 3 for each treatments.
