# Supplementary figures and images for: Beta‐amyloid 1‐42 monomers, but not oligomers, produce PHF‐like conformation of Tau protein
Source: Aging Cell. 2016 Jul 12;15(5):914–23. doi: 10.1111/acel.12500 (PMC5013016; doi:10.1111/acel.12500)

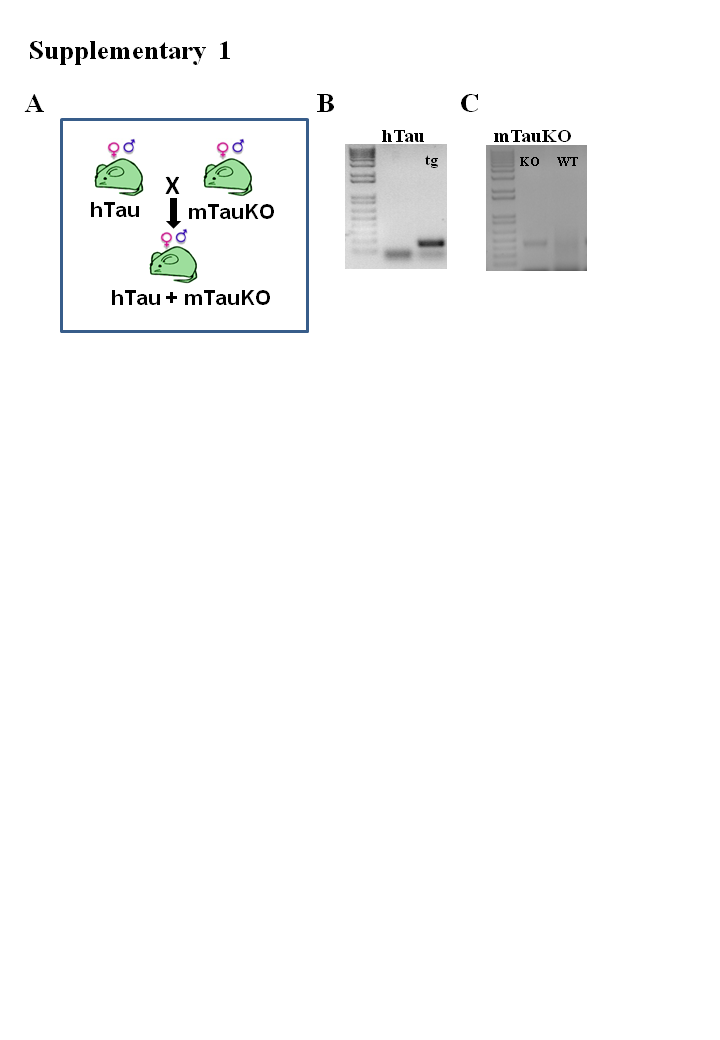

Supplement: Supplementary file 1 — Fig. S1 (A) Breeding scheme from mating hTau mice (Mapt tm1(EGFP)KltTg(MAPT) 8cPdav/J; #004808, Jackson Laboratory); murine (m) Tau knock‐out (KO) mice (Mapt tm1(EGFP)Klt/J; #004779, Jackson Laboratory) to generate hTau + mTauKO mice. (B, C) Specific PCR analysis of genomic DNA. Note: (B) transgene (tg) = 187 bp. (c) Mutant (KO) = 490 bp. 1Kb DNA ledder was used in both analysis [file ACEL-15-914-s001.tif]

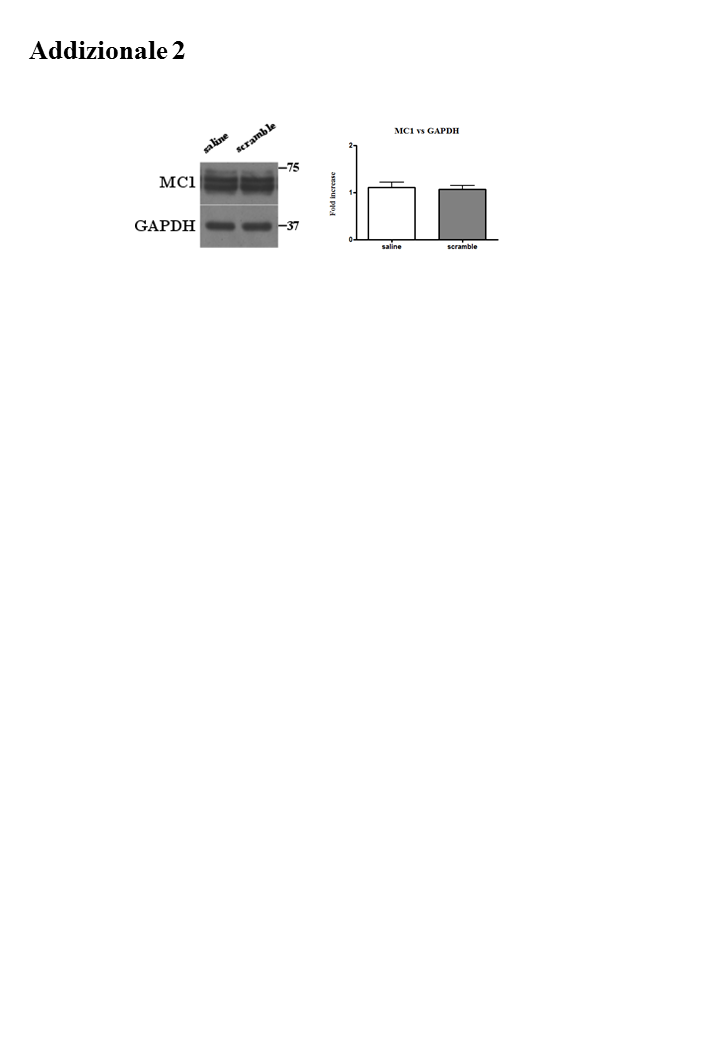

Supplement: Supplementary file 2 — Fig. S2 Representative western‐blot of brain extracts from control (saline) and mice injected with Aβ 1‐42 scramble preparation using MC1 (A) antibody for detection. Densitometric quantification did not reveal changes in the total protein level of MC1 induced by both treatments. An antibody raised against GAPDH served as loading control. n = 3 for each treatments. [file ACEL-15-914-s002.TIF]
