# Supplementary material for: Beta‐amyloid 1‐42 monomers, but not oligomers, produce PHF‐like conformation of Tau protein
Source: Aging Cell. 2016 Jul 12;15(5):914–23. doi: 10.1111/acel.12500 (PMC5013016; doi:10.1111/acel.12500)
Supplement: Supplementary file 3 [file ACEL-15-914-s003.docx]

**Supplementary Legends**

**Supplementary legend 1**

(A) Breeding scheme from mating hTau mice (Mapt ^tm1(EGFP)Klt^Tg(MAPT) 8cPdav/J; #004808, Jackson Laboratory)*;* murine (m) Tau knock-out (KO) mice (Mapt ^tm1(EGFP)Klt^/J; #004779, Jackson Laboratory) to generate hTau + mTauKO mice. (B-C) Specific PCR analysis of genomic DNA. Note: (B) transgene (tg) = 187 bp. (c) Mutant (KO) = 490 bp. 1Kb DNA ledder was used in both analysis.

**Supplementary legend 2**

Representative western-blot of brain extracts from control (saline) and mice injected with Aβ 1-42 scramble preparation using MC1 (**A**) antibody for detection. Densitometric quantification did not reveal changes in the total protein level of MC1 induced by both treatments. An antibody raised against GAPDH served as loading control. n=3 for each treatments.
